# Long-term immunity induced by SPBN GASGAS in orally vaccinated dogs is non-inferior to inactivated rabies vaccines

**DOI:** 10.1016/j.jvacx.2023.100410

**Published:** 2023-11-21

**Authors:** Ad Vos, Suwicha Kasemsuwan, Kansuda Leelahapongsathon, Katharina Bobe, David Perez-Bravo, Jeannette Kliemt, Parinya Phawaphutayanchi, Nirut Aiyara, Conrad M. Freuling, Thomas Müller

**Affiliations:** a Ceva Innovation Center, 06861 Dessau–Rosslau, Germany; b Faculty of Veterinary Medicine, Kasetsart University, Bangkok 10900, Thailand; c Institute of Molecular Virology and Cell Biology, Friedrich-Loeffler-Institut (FLI), WHO Collaborating Centre for Rabies Surveillance and Research, 17493 Greifswald-Insel Riems, Germany; d Department of Health, Bangkok Metropolitan, Thapthan 61120, Thailand

**Keywords:** Oral vaccination, Dog, SPBN GASGAS, DOI, Immunobridging, Efficacy

## Abstract

In a long-term immunogenicity study (1100 days post vaccination) in local Thai dogs the immune response of the oral rabies vaccine SPBN GASGAS was compared to those elicited by a commercial inactivated vaccine using immunobridging. Based on the detection of rabies virus binding (rVBA) and rabies virus neutralizing antibodies (rVNA) as measured by ELISA and Rapid Fluorescent Focus Inhibition Test (RFFIT) the long-term immune response in dogs vaccinated orally with the SPBNA GASGAS strain of rabies vaccine in a bait was non-inferior to a conventional inactivated rabies vaccine. The outcome of this study supports extending the originally claimed duration of immunity (DOI) of SPBN GASGAS after oral vaccination for dogs from 6 to 30 months.

## Introduction

The duration of immunity (DOI), sometimes also called duration of protection, is an essential part of efficacy studies whereby efficacy here means induction of protective immunity [1]. The claimed DOI is most of the time determined for the minimum specified duration because of the costs and animal welfare implications of such studies [2]. DOI studies involving rabies virus challenge infection for oral rabies vaccines for dogs meeting international requirements are rare. Most studies included only a limited number of animals, a short observation period (<3 months), or animals (partially) received the vaccine by direct oral instillation instead of being offered a bait [3], [4], [5], [6], [7]. While the European Union (EU) regulatory requirements provide standardized procedures for evaluating vaccine efficacy for a number of diseases, however, presently, the only monograph in the European Pharmacopoeia with specific requirements to establish a DOI is for rabies vaccines [2].

The efficacy of the oral rabies virus vaccine strain, SPBN GASGAS, was shown in dogs upon an experimental rabies challenge infection, hence, the DOI of the oral rabies vaccines SPBN GASGAS for dogs was determined using the minimum required number of animals and minimum specified duration between vaccination and challenge (180 days) [8]. However, results on long-term protective immunity of this vaccine construct in another target species, the red fox (*Vulpes vulpes*), indicated that these animals were protected for a much longer period of time [9]. The regulatory authorities acknowledge that DOI-studies, including a challenge infection, are not only expensive and time-consuming but most of all are associated with animal welfare issues [2]. To limit the impact and number of challenge studies, it is suggested to measure protection using a suitable indicator other than challenge infection. For an indicator to be acceptable, evidence must be provided that there is sufficient relationship between the indicator and protective immunity in the target species against the disease concerned [1], [2]. Hence, once vaccine efficacy has been established under the required conditions, immunobridging can be used to extrapolate the likelihood of a vaccine’s protective effect by converting immunogenicity to vaccine efficacy estimates [10] such as evaluating an extension of DOI.

Differences in immune response between animals kept under experimental or field conditions have been observed, whereby vaccines generally tend to perform better in experimental animals [11]. Research animals are typically in optimal health, well fed using a balanced nutritional diet, have received core vaccinations and are often treated preventively against endo- and ectoparasites. Furthermore, animals that do not respond to a vaccine can be recognized as such and accounted for in the analysis. In contrast, a local dog population consists of animals with varying ages, different breeds, health status and with highly variable quantitative and qualitative food availability. These factors can affect the initial response to a vaccine as well as later resistance to disease [12], [13]. For example, poor nutrition can suppress immune responses by decreasing nutrient availability for protein (e.g. antibody and cytokine) synthesis [13]. Consequently, also immunogenicity studies with local dogs from canine rabies endemic countries have been performed with SPBN GASGAS [14], [15], [16]. In most of these studies, blood sampling was done within the first 2 months post vaccination. Only in Morocco, blood samples from dogs were collected up to 3 years post vaccination (unpublished). Long-term immunogenicity studies under field conditions are difficult as the drop-out rate is often very high due to high death rate [17] or being unable to relocate the dogs during subsequent visits. For example, 12.4 % of the dogs included in an immunogenicity study died during the first month post vaccination in Bali – Indonesia [16]. In Haiti, the drop-out rate was even higher; already 27 % of the dogs were not available for post vaccination blood sampling 17 dpv [14]. Therefore, it was decided to conduct a semi-controlled immunogenicity study with SPBN GASGAS in local dogs kept in a Thai dog shelter [18]. Here, a similar immune response was observed in local dogs as in the experimental challenge study [8] with a detectable immune response (ELISA) in orally vaccinated dogs for at least 12 months after being offered a vaccine bait [18].

To obtain more information on the duration of the detectable humoral immune response in dogs after oral vaccination in terms of rabies virus neutralizing antibodies (rVNA) and rabies virus binding antibodies (rVBA), it was decided to prolongate the study and examine the long-term immunity of SPBN GASGAS in more detail by using immunobridging.

## Material & methods

The study design including animals, housing and feeding conditions, the sampling and testing procedures and diagnostic assays applied are described in detail in a previous study [18]. Briefly, a total of 46 young local dogs (26 males and 20 females) were kept at the study site, Bangkok Metropolitan Administration’s dog shelter in Taptan, Uthai-Thani province, Thailand. The dogs had never been exposed to rabies virus and rabies vaccination prior to this study. Prior to the actual study, animals were vaccinated with a combination vaccine (RECOMBITEK® C8, Merial (Thailand) Ltd., Bangkok, Thailand) against canine distemper, parvovirus infection, adenovirus infection, bronchitis, and leptospirosis and treated with anthelminthics.

The dogs were randomly allocated into 5 treatment groups: Group A—dogs receiving a boiled pig intestine bait segment in which a PVC-sachet with aluminum cover foil containing the liquid vaccine was incorporated (bait, n = 15), Group B—dogs receiving the vaccine by direct oral administration (d.o.a., n = 10), Group C—dogs vaccinated by the parenteral route (s.c., n = 10), Group D—dogs receiving a placebo intestine bait and kept in the same cage as vaccinated dogs (placebo, n = 7), and Group E—non-vaccinated naïve control dogs (control, n = 4). Two dogs were caged together, except for 2 control dogs that were kept individually. Dogs were aged on average 7 months (range: 3 – 12 months) at the day of primary vaccination, making sure that possible maternally derived antibodies did not interfere with vaccination.

For oral vaccination, the vaccine strain SPBN GASGAS was used, a highly attenuated, stable derivative of a cDNA clone (SAD L16) of the vaccine strain SAD B19 genetically modified by site-directed mutagenesis [8], [19]. The bait offered to the dogs in Group A contained a sachet filled with 3.0 mL SPBN GASGAS (10^8.2^ FFU/mL). For Group B, 3.0 mL of vaccine virus with the same titre was slowly released into the oral cavity of the dog using a needleless syringe. For dogs vaccinated by the parenteral route (s.c.), a commercial locally available product (Bayovac*R, Bayer Thai Co. Ltd., Bangkok Thailand) was used.

Dogs were tested for the presence of antibodies against rabies 7 days before vaccination (B0) to ensure their naïve status for rabies antibody (ELISA). Initially, blood samples were collected from all animals on 7 (B1), 14 (B2), 28 (B3), 90 (B4), 180 (B5) and 365 (B6) [18]. In the frame of this study, additional blood samples were taken at 545 (B7), 735 (B8), 916 (B9) and 1100 (B10) days post-vaccination (dpv). The control and placebo animals (Group D&E) received their primary vaccination directly after collection of the B6-sample, 365 dpv. Unintentionally, all animals were boostered with an inactivated rabies vaccine (s.c.) approximately one month prior to the planned end of the study (B10).

After collection of blood samples, serum was separated after blood clotting at refrigerated temperature for 24 h and removed by centrifuging at 1,000–2,000 x g for 10 min in a refrigerated centrifuge and stored at − 20 ^◦^C until testing. The study was approved by Kasetsart University’s Institutional Animal Care and Use Committee (ACKU 61-VET-011). The presence and level of rVNA and rVBA was determined for all samples taken using a commercial blocking ELISA (O.K. Servis BioPro, Prague, Czech Republic) according to the manufacturers’ instructions [20] and a modified Rapid Fluorescent Focus Inhibition Test (RFFIT) using the challenge virus standard (CVS-11) as a test virus with titers expressed in IU/mL [21], respectively. To ensure the comparability of results, the cut-off for seropositivity was set at ≥ 40 % inhibition for ELISA as per manufacturers instruction and to ≥ 0.5 IU/mL for RFFIT, as in the original study [18]. The seroconversion rate was used to indicate the proportion of samples that met the specified threshold. Differences in individual rVBA and rVNA values between treatment groups at different sampling time points were tested for significance using unpaired T-tests with a significance level of α = 0.05. All statistical analyses were carried out using GraphPad Prism 7 (GraphPad Software Inc., San Diego, CA, USA).

## Results

**Fig. 1:**
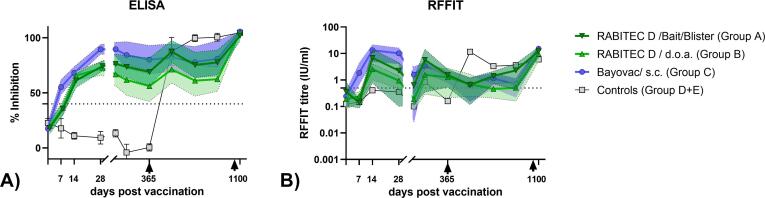
Post vaccination mean Percentage Blocking (%) values and Geometric Mean Titres (IU/ml) and resulting 95% confidence intervals of the different treatment groups as determined by ELISA (A) and RFFIT (y-axis is logarithmically transformed) (B).

Dogs vaccinated via the oral route with SPBN GASGAS (Groups A&B) mounted a measurable immune response, with a slight delay as compared to parenterally vaccinated animals (Fig. 1A and B). For the ELISA, irrespective of treatment, the mean PB-values of all vaccinated groups were above the threshold of seropositivity of 40 % during the entire observation period (Fig. 1A).

While during the first 90 dpv, at most sampling points (7, 28 & 90 dpv) dogs that consumed a vaccine bait had significantly lower rVBA levels than dogs vaccinated parenterally (s.c.); (unpaired t-Test, 2-sided), no significant difference was observed in the level of rVBA between these two groups from 180 dpv onwards (Table 1). The group of control and placebo animals tested seronegative and seropositive before and after the primary vaccination on day 365, respectively, except for one placebo animal that tested positive in ELISA 7dpv. The obtained value (43 %) for this particular sample was very close to the cut-off value of 40 % and the subsequent 5 pre-vaccination serum samples taken from this animal were all below the seropositivity threshold. The booster effect at the end of the study in the vaccinated animals is also clearly visible. A similar pattern can be observed with the RFFIT-results. However, here GMT-values at or below the threshold (0.5 IU/mL) can be observed (Fig. 1B). Individual data sets for ELISA and RFFIT are provided in the supplement (supplementary table 1).

**Table 1:** Results (p-values) of statistical comparative analysis (unpaired t-Test, 2-sided) of rVBA (ELISA) and rVNA (RFFIT) values of the individual dogs between different treatment groups [bait, d.o.a., s.c. (parenteral), oral – bait & d.o.a. pooled] for each sampling time point (B0-10).

**Blood- sample**		**bait (group A) vs. d.o.a. (group B)**			**oral (groups A + B) vs. parenteral (group C)**			**bait (group B) vs. parenteral (group C)**
	**dpv**	**ELISA**	**RFFIT**		**ELISA**	**RFFIT**	**ELISA**	**RFFIT**
B0	−7	0.68	0.54		0.63	0.83	0.81	0.68
B1	7	0.81	0.04		**<0.0001**	**0.02**	**<0.0001**	0.07
B2	14	0.28	0.34		0.15	0.06	0.12	0.23
B3	28	0.63	0.35		**<0.0001**	**0.0002**	**<0.0001**	**0.005**
B4	90	0.15	0.15		**0.003**	**0.03**	**0.03**	0.15
B5	180	0.13	0.41		**0.03**	0.85	0.15	0.63
B6	365	0.15	0.74		**0.05**	0.91	0.21	0.96
B7	545	0.03*	0.94		0.83	0.73	0.46	0.69
B8	735	0.1	**0.03**		0.28	0.75	0.76	0.56
B9	916	0.05*	0.08		0.20	0.32	0.70	0.14
B10	1100	0.29	0.47		0.26	0.20	0.20	0.41

* - the mean value for dogs offered a bait was higher than d.o.a. treated animals, p-values ≤ 0.5 are highlighted in bold.

At 28 dpv, all vaccinated dogs, irrespective of treatment, seroconverted using the ELISA. Subsequently, seroconversion rate using ELISA remained high during the entire observation period. The seroconversion rate in the orally vaccinated animals (Group A: bait and Group B: d.o.a.) based on the RFFIT results never reached 100 %. Meanwhile, all dogs vaccinated by the parenteral route tested seropositive for rVNA between 14 and 365 dpv except 90 dpv. The seroconversion rate based on rVNA (RFFIT) showed much more temporal fluctuations than the seroconversion rate of rVBA (ELISA) (Fig. 1B).

Based on ELISA data more than 90 % of the orally vaccinated dogs (both groups A + B pooled) seroconverted on each sampling point after vaccination and prior to booster vaccination except for 7 (B1) and 365 (B6) dpv (Table 2).

**Table 2:** Comparison of seroconversion rate (%), incl. upper and lower limit of 95% confidence intervals (CI) based on ELISA-results using 40% PB as cut-off between parenterally and orally vaccinated dogs [Group A (bait) and B (d.o.a.) pooled] prior to booster vaccination.

**Blood sampling**	**B0**	**B1**	**B2**	**B3**	**B4**	**B5**	**B6**	**B7**	**B8**	**B9**
	Inactivated vaccine (group C)									
Number of animals	10	10	10	10	10	10	10	10	9	9
% seroconversion	0	100	100	100	100	100	90.0	100	90.0	88.8
Upper 95 %CI	30.9	100	100	100	100	100	100	100	100	100
Lower 95 %CI	0	69.1	69.1	69.1	69.1	69.1	55.4	69.1	55.4	50.7
	SPBN GASGAS (groups A + B)									
Number of animals	25	25	25	25	25	25	25	23*^#^	23*^#^	24*
% seroconversion	0	32.0	96.0	100	96.0	92.0	84.0	100	91.7	95.8
Upper 95 %CI	13.0	51.6	99.8	100	99.8	98.6	93.6	100	98.5	99.8
Lower 95 %CI	0	17.2	80.5	86.7	80.5	75.0	65.3	85.7	74.2	79.8

* one animal in group A died of reasons unknown; ^#^ no blood sample could be taken from another animal.

While overall test agreement (kappa) between ELISA and RFFIT across all treatment groups and sampling time points was 0.438 (95 % confidence interval: 0.533–0.677), test agreement during the period up to one year (kappa 0.541; 95 % confidence interval: 0.431–0.597) was considerably higher compared to the period more than one year (kappa 0.036; 95 % confidence interval: −0.036–0.108) (supplementary table 1).

## Discussion

At present, rabies vaccines are the only products for which the regulatory authorities in the US or EU require minimum DOI studies for licensing purposes [2], [22]. Based on challenge studies, the minimum DOI for several rabies vaccines is 3 years and based on serologic studies some inactivated vaccines were shown to have a minimum DOI of 5–7 years [22], [23]. In this study, we used immunobridging as an alternative to conventional efficacy studies [10] by adhering to the 3R concept of animal welfare and demonstrated a comparable immune response of the oral vaccine strain SPBN GASGAS in dogs with those of an approved inactivated vaccine. Because of the protective correlates of immunity, the long-term protective effect of SPBN GASGAS in dogs can be inferred by this study, supporting the extension of the established DOI. Immunobridging studies become standard in human medicine and are intended to help stimulate continued vaccine development while ensuring appropriate assessment of safety and efficacy. Those studies can be sufficient to ascertain immunological non-inferiority for licensure for alternate vaccines, with post-licensure surveillance confirming effectiveness as shown for papillomavirus, Ebola virus and SARS-CoV-2 vaccines [24], [25], [26].

**Fig. 2:**
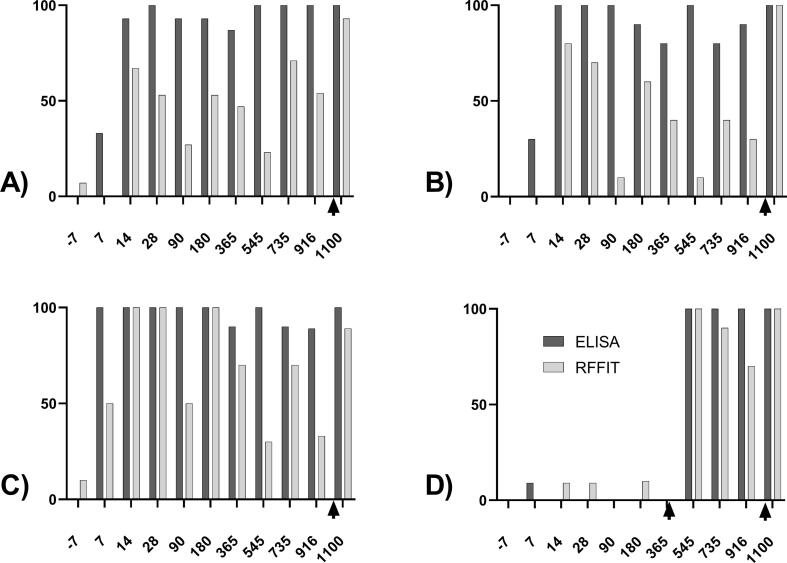
Percentage of seroconverted dogs (≥40 % PB in ELISA, ≥0.5 IU/ml in RFFIT) in different treatment groups at individual sampling time points; A) after vaccination via bait (group A), B) via direct oral application (d.o.a.; group B), C) via parenteral vaccination (group C) compared to control animals (D, groups D + E). Additional parenteral booster vaccinations are indicated (black arrows).

It is supposed that vaccination with modified rabies virus vaccines mimics natural infections with the infectious agent and therefore is believed to induce stronger and longer-lasting immunity compared to inactivated replication-deficient vaccines [13], [22]. Based on the results presented in this study, there was no difference in seroconversion rates and rVBA levels as of day 180 pv between orally vaccinated dogs with SPBN GASGAS and those vaccinated with an approved inactivated rabies vaccine (Table 1, Table 2, Fig. 2), suggesting that the long-lasting immune response induced by SPBN GASGAS is non-inferior. Therefore, it seems justified to claim that the originally reported DOI of SPBN GASGAS in dogs of 6 months persists much longer (>30 months), as has been shown for several inactivated rabies vaccines for domestic and farm animals [13], [27], [28]. There is evidence, however, that protection of oral rabies vaccines including SPBN GASGAS can even last longer (between 36 and 83 months post vaccination) as shown in captive foxes after oral vaccination with high-dosed attenuated oral rabies vaccines [9], [29].

Antibody is a primary mechanism of protective immunity for the canine vaccines including CDV, CPV-2, CAV-1, and rabies [30]. During previous challenge studies it was shown that the sero-response rate using the commercial competition ELISA for orally vaccinated animals was a better indicator for the sero-protection rate than rates obtained by using the RFFIT. Here, the 40 % inhibition threshold was found to be an excellent predictor of survival for orally vaccinated species like red fox, raccoon dog, mongoose, and dog; with 90 % or more animals with rBNA levels above this cut-off surviving challenge [8], [9], [21], [31]. In the present study, we confirm that also long-term humoral immunity can be better followed using the BioPro ELISA than the RFFIT [9]. While the overall test agreement (kappa) between ELISA and RFFIT across all treatment groups and sampling time points as well as in the period up to one year was considered moderate, there was only slight agreement between the two tests in the period more than one year. One reason is that the RFFIT seems to be more sensitive to variations during test performance than the ELISA. For example, the observed drop in GMT (RFFIT) at 90 dpv was observed in all groups, incl. the control and placebo group (Fig. 1). Often rVNAs above a certain threshold (0.5 IU/mL) are considered as the most relevant indicator of protective immunity [32], [33]. However, vaccinated dogs without detectable or low rVNAs (<0.5 IU/mL) can still be protected against RABV infection and vice versa, dogs with rVNA > 0.5 IU/mL can succumb to rabies [5], [8], [23], [34]. Even if 70 % inhibition as per the manufacturer’s instructions would be used to equal 0.5 IU/mL, it does not result in a better test agreement, which shows that the adjustment of the cut-offs of an ELISA to match results obtained in serum neutralization tests is not expedient. Immunity to rabies is a complex process resulting from various combinations of humoral and cell-mediated immunity, and interactions between innate and adaptive immune responses. Previous DOI studies demonstrated that immunologic memory can exist even in vaccinated dogs with serum antibody titer < 0.1 IU/mL [28]. However, an increase in sensitivity in seroneutralisation tests comes at the expense of specificity if cut-off value is lowered. In RFFIT or FAVN, it becomes increasingly difficult to distinguish between specific and non-specific neutralising activity at lower cut-off values. Thus, the debate remains if there is a ubiquitous cut-off level of rVNAs as being invariably (>90 %) protective.

In contrast, the 40 % cut-off of the BioPro ELISA seems to be suitable as surrogate for protective immunity for oral vaccination of dogs with SPBN GASGAS as shown in two previous studies [8], [21]. Here, in the first study, a total of 16 vaccinated and 4 control dogs were challenged 56 days, while in the other study, 25 vaccinated and 10 control dogs were inoculated with RABV 180 days post vaccination. All 14 control animals tested seronegative on the day of challenge and subsequently all succumbed to infection. In contrast, all 41 vaccinated dogs were seropositive in ELISA prior to challenge infection and all but one (2.4 %) remained healthy 90 days post challenge [8], [21]. Certainly, challenge infection would be the ultimate proof of the long-term protective effect of the induced immune response in dogs after oral vaccination with this vaccine construct, which could be considered a limitation of this study. According to the European Pharmacopoeia and WOAH regulations at least 92 % and 88 % of vaccinated dogs must survive the challenge infection, respectively [35], [36]. On the one hand, long-term efficacy of SPBN GASGAS has already been demonstrated in red foxes [37]. On the other hand, the results obtained with the blocking ELISA used and the currently defined cut-off of 40 % inhibition in dogs offers sufficient evidence to be a very good predictor (>90 %) of survivorship [21] during a relevant challenge infection in alignment with the Note for Guidance on duration of protection achieved by veterinary vaccine by the Committee for Medicinal Products for Veterinary Use (CVMP) of the EMA s [1].

Although the disappearance of circulating rabies specific antibodies makes it difficult to determine if there is still protective immunity, a booster vaccination can be used to detect an accelerated anamnestic response, as after challenge infection. A rapid appearance or rise in antibody levels is considered an indication that memory cells still exist when antibodies were absent or present at low levels, respectively [28]. Although in this study all dogs were still seropositive at 30 months post vaccination, the pronounced anamnestic reaction observed after booster vaccination approximately 35 months post primary vaccination (Fig. 2) is indicative of a long-term memory response after oral vaccination with SPBN GASGAS. Hence, rVNA and rVBA can be considered accurate and acceptable correlates of efficacy/vaccine protection. Considering the relatively short lifespan of free-roaming dogs in most countries with dog-mediated rabies [17], [38], [39], [40], [41], [42], the consumption of a single vaccine bait containing SPBN GASGAS will under most circumstances induce lifelong protection.

## Conclusions

Regulatory authorities acknowledge the animal welfare implications associated with efficacy studies, including determining the DOI. We have shown that for the oral rabies vaccine SPBN GASGAS and the induced humoral immune response as measured by this particular ELISA, the 40 % threshold is acceptable as a suitable indicator for protective immunity after oral vaccination in target species like the domestic dog. Based on the outcome of this study it seems therefore warranted extending the originally claimed DOI of SPBN GASGAS after oral vaccination for dogs from 6 to 30 months.

## Funding Source Declaration

This study was partly funded by Her Royal Highness Princess Chulabhorn Mahidol in the frame of the “Saving Animals and Human Lives from Rabies” Project as well as by IDT Biologika, Germany / Ceva Santé Animale, France, for investigating immune mechanisms and response to oral rabies vaccination of reservoir species.

## CRediT authorship contribution statement

**Ad Vos:** Formal analysis, Conceptualization. **Suwicha Kasemsuwan:** Project administration, Resources, Conceptualization. **Kansuda Leelahapongsathon:** Project administration, Resources, Funding acquisition. **Katharina Bobe:** Formal analysis, Data curation, Supervision. **David Perez-Bravo:** Investigation, Formal analysis. **Jeannette Kliemt:** Investigation, Methodology. **Parinya Phawaphutayanchi:** Supervision, Resources. **Nirut Aiyara:** Supervision, Resources. **Conrad M. Freuling:** Investigation, Software, Validation, Visualization. **Thomas Müller:** Investigation, Writing – original draft, Writing – review & editing.

## Declaration of Competing Interest

The authors declare the following financial interests/personal relationships which may be considered as potential competing interests: AV, KB, and DPB are full-time employees of Ceva Innovation Center, formerly IDT Biologika, Germany, a company manufacturing oral rabies vaccine baits. T.M. and C.M.F. from the Friedrich-Loeffler-Institute, Germany, received funding from IDT Biologika / Ceva for research into mechanisms and immune response of oral rabies vaccination. All other authors declare that there is no financial/personal interest or belief, or intellectual property that could affect their objectivity and that no competing interests exist.

## Data Availability

All data are included in the manuscript.
